# Successful Coil Embolization of Active Bleeding From a Replaced Left Hepatic Artery to the Left Gastric Artery Associated With a Traumatic Rupture of a Simple Hepatic Cyst Causing Hemodynamic Instability

**DOI:** 10.7759/cureus.60907

**Published:** 2024-05-23

**Authors:** Saptarshi Biswas, Kaitlyn Spinella, Danielle N Lang

**Affiliations:** 1 Surgery, Grand Strand Medical Center, Myrtle Beach, USA; 2 Medicine, Lake Erie College of Osteopathic Medicine, Erie, USA; 3 General Surgery, Forbes Hospital, Allegheny Health Network, Erie, USA

**Keywords:** traumatic repair, polycystic liver disease, intra-abdominal hemorrhage

## Abstract

Intra-abdominal hemorrhage resulting from a ruptured, large hepatic cyst in a polycystic liver disease (PCLD) patient is rare and potentially fatal if not addressed promptly. Only a few isolated cases have previously been reported. The usual patient profile consists of elderly patients on anticoagulation, as is demonstrated in our case. Intra-hepatic cysts are broadly classified into congenital, traumatic, infectious, parasitic, and neoplastic. Congenital intra-hepatic cysts can consist of both simple and PCLD, as is outlined in our case. Simple cysts are usually asymptomatic, but occasionally they may achieve larger dimensions and lead to complications such as rupture, obstruction, infection, hemorrhage, and even portal hypertension.

We present an uncommon case of a 78-year-old patient with PCLD on rivaroxaban who presented initially with diffuse abdominal pain, distension, and progression into hemodynamic instability. A computerized tomography (CT) scan revealed a ruptured left hepatic lobe cyst, causing hemoperitoneum and resulting in an acute abdomen. This case was complicated by the patient’s anticoagulation status and anomalous hepatic vasculature pattern. Interventional radiology (IR) successfully identified the aberrant bleeding vessel and stopped the active extravasation with super-selective coil embolization.

## Introduction

In the general population, the frequency of hepatic cyst formation is reported to be between 1.6%-3.6% [1]. Polycystic liver disease (PCLD) is a condition resulting from the impairment of biliary ductal cell development during embryology [2]. Since the condition is typically asymptomatic, the cysts are usually identified incidentally on imaging. These cysts can be complicated by compression, obstructive jaundice, portal hypertension, infection, and intra-cystic bleeding [3-9]. However, one exceedingly rare complication of hepatic cysts is traumatic hemorrhagic rupture. This complication has only been minimally described in the literature, where only isolated cases of fatal bleeding have been reported [10]. There is a general lack of consensus on the most appropriate treatment guidelines due to the minimal number of cases reported.

We present a rare case of an elderly, anticoagulated patient who suffered a fall that resulted in the rupture of a large hepatic cyst. Initially, he presented with diffuse abdominal pain and progressive distension, ultimately leading to hemodynamic instability. This case was further complicated by the patient’s anticoagulation status and anomalous hepatic vasculature pattern. Interventional radiology (IR) successfully identified the aberrant bleeding vessel and stopped the active extravasation with super-selective coil embolization.

## Case presentation

A 78-year-old male presented to the emergency department (ED) for evaluation of abdominal pain following a syncopal episode. According to the patient, he got up in the morning to go to the bathroom when he collapsed to the ground because he “felt very dizzy." The patient last remembers grasping onto a railing when falling to the ground. He believes he was “out for a couple of seconds” and incontinent of urine. He complained of abdominal pain, describing it as moderate-to-severe in intensity, diffuse in distribution, and associated with increasing abdominal distention. Although he admitted to nausea, he denied any chest pain, shortness of breath, vomiting, or diarrhea.

The patient was seen in the ED the week prior for abdominal pain. His workup at that time included routine labs and a CT scan of his abdomen, which showed numerous hepatic cysts, some of which were calcified, representing benign polycystic liver disease. The largest cyst measured 15 centimeters from the left hepatic lobe and contained hyperdense material consistent with hemorrhage. A right common iliac artery stent was noted. A structure measuring approximately 5 cm in the right hemipelvis with adjacent postsurgical changes was identified, most likely representing an internal iliac artery aneurysm and mild, non-specific bladder wall thickening. The patient was discharged from the ED following this evaluation.

A review of the systems revealed a patient who was well-developed, well-nourished, non-toxic appearing, and oriented to person, place, and time. The physical examination was essentially negative except for his abdominal findings. His abdominal examination revealed guarding, distension, and diffusely distributed tenderness on palpation. The differential diagnoses included the possibility of a cardiac event, perforated viscus, pulmonary embolism, obstructive process, and urosepsis.

His medical history was comprised of polycystic liver disease, atrial fibrillation status post-ablation, hyperlipidemia, hypertension, and obstructive sleep apnea on continuous positive airway pressure (CPAP). His surgical history consisted of cardiac catheterization, cardioversion for atrial flutter, cataract extraction, cholecystectomy, operative repair of a boxer’s fracture, and an iliac artery stent.

His current medications included alprazolam, atorvastatin, diltiazem, omeprazole, rivaroxaban, sertraline, acetaminophen, apixaban, and phytonadione. He reported allergic reactions (rash) to amoxicillin, penicillin, and tetracycline. The patient was anticoagulated with rivaroxaban for his atrial fibrillation. His family history consisted of arthritis, stroke (mother), and heart failure (father).

His vital signs on presentation were as follows: blood pressure of 118/75; pulse rate of 90 beats per minute; temperature of 98.4°F; respiratory rate of 18; peripheral capillary oxygen saturation (SpO2) of 96% on room air. The labs were as follows: red blood cells: 2.81 cells/mcL; hemoglobin: 8.6 g/dl; hematocrit: 26.0%; mean corpuscular hemoglobin concentration (MCHC): 33.1 g/dl; and neutrophils: 86%. His comprehensive metabolic panel (CMP) results were as follows: glucose: 146 mg/dL; creatinine: 0.63 mg/dL; and alkaline phosphatase: 164 U/L. A coagulation profile revealed the following: activated partial thromboplastin time (aPTT) of 52 seconds; international normalized ratio (INR) of 4.2. His troponins, liver function tests, and lipase were within normal limits.

**Figure 1:**
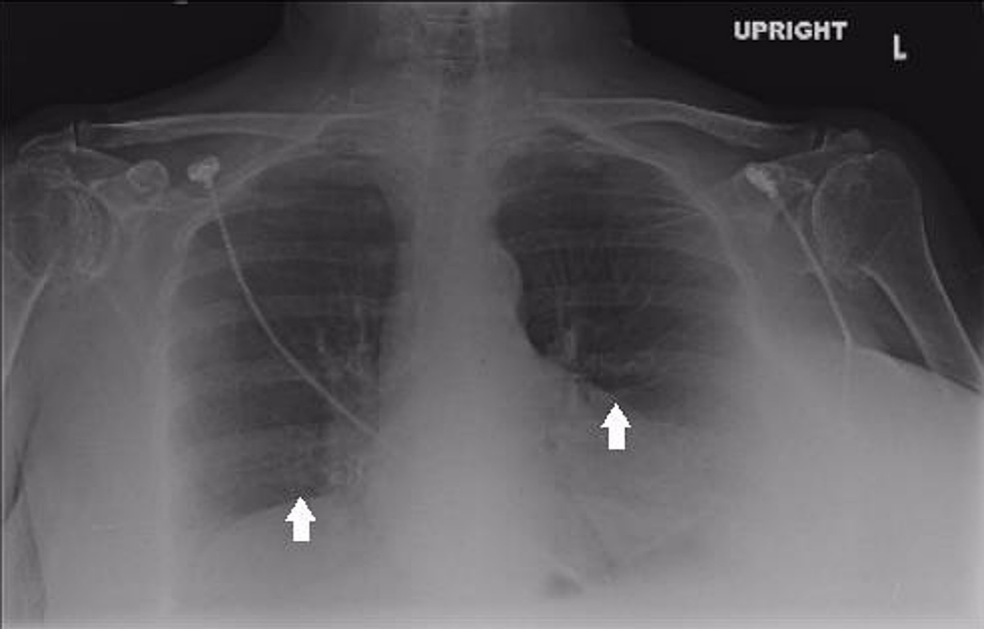
The chest X-ray shows shallow lung volumes with minor bibasilar atelectasis.

**Figure 2:**
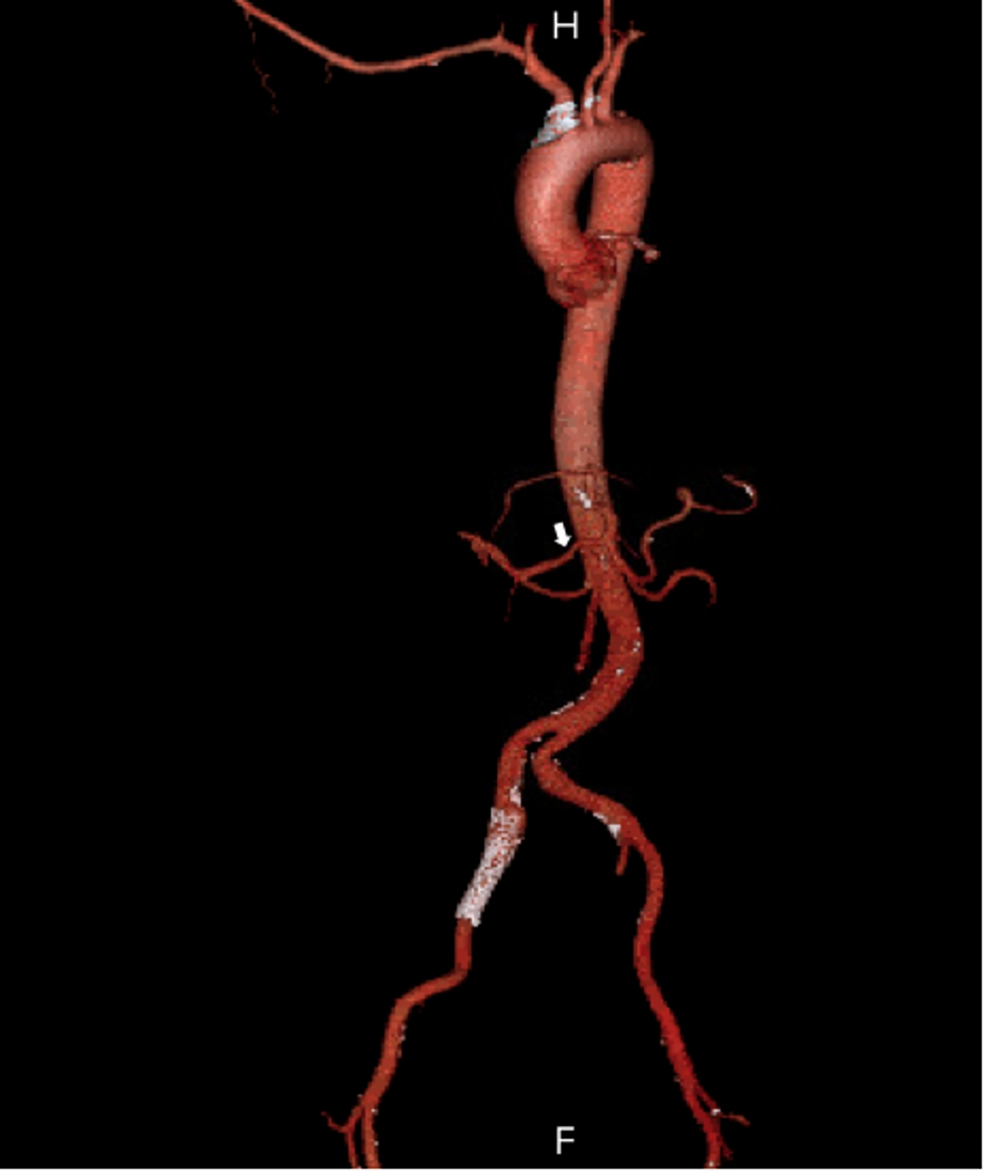
A CT angiogram demonstrates a mildly narrowed celiac axis with a near-completely replaced hepatic artery off of the superior mesenteric artery (SMA) and an accessory left hepatic artery off of the left gastric artery (normal variant).

Chest X-rays (Figure 1) showed shallow lung volumes with minor bibasilar atelectasis. A computerized tomography angiogram (CTA) of the chest, abdomen, and pelvis utilizing an aortic protocol with three-dimensional (3D) volumetric rendered images was obtained. No evidence of dissection or aneurysms was noted. The great vessels originated in a normal sequential manner. The celiac axis was mildly narrowed at its origin, with a near-completely replaced hepatic artery off the superior mesenteric artery (SMA) and an accessory left hepatic artery arising off the left gastric artery (Figure 2). There was a single right renal artery but two left renal arteries. A right common iliac artery stent with minimal contrast was present, extending laterally to its origin. There was a thrombosed aneurysm of the right internal iliac artery.

**Figure 3:**
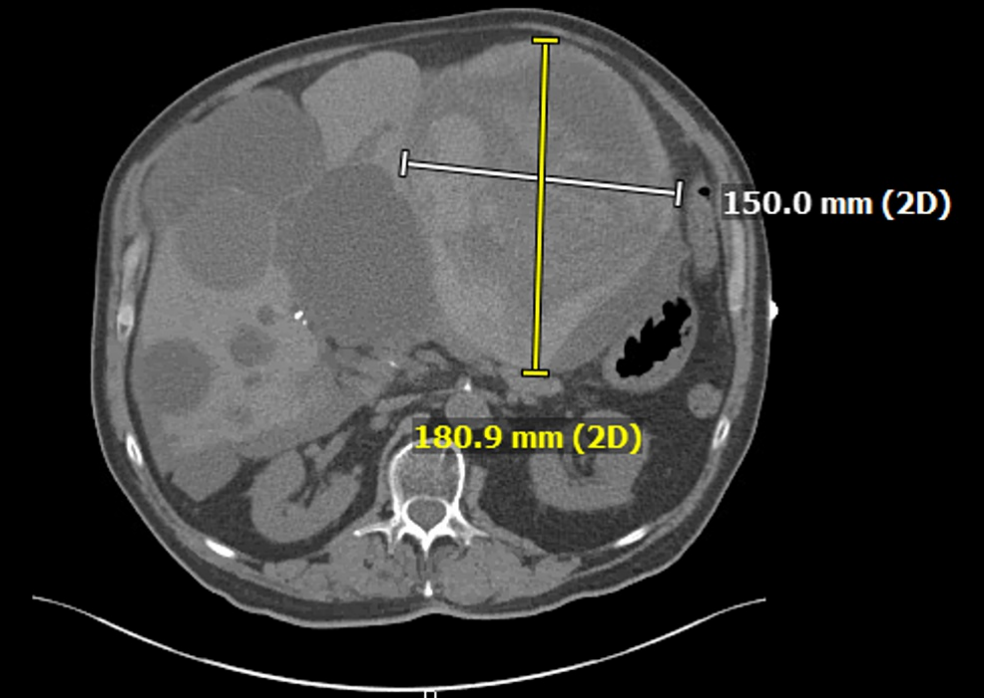
An axial view of the left hepatic cyst, measuring 18.9x15cm, shows the interval progression of hemorrhage within the lesion.

**Figure 4:**
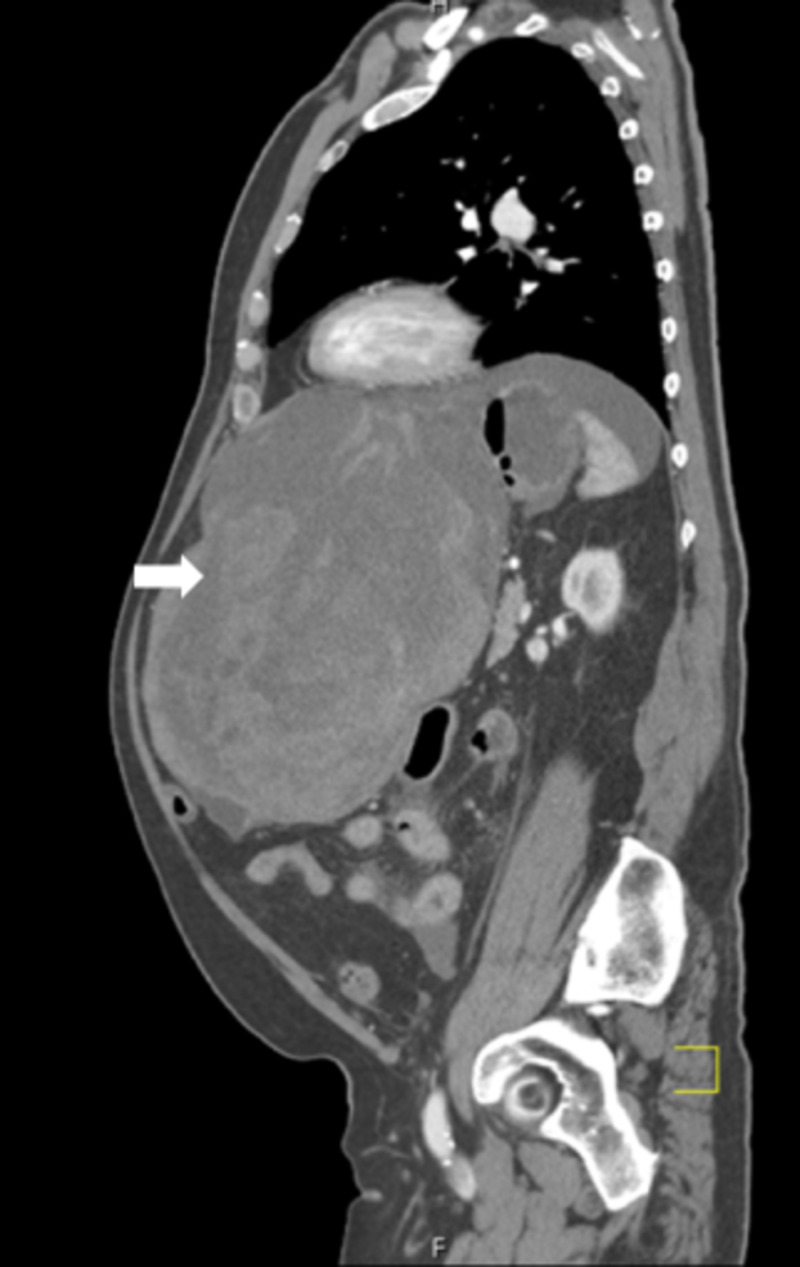
The sagittal view of the left hepatic cyst shows an interval progression of hemorrhage within the lesion, demonstrating significant abdominal distention.

**Figure 5:**
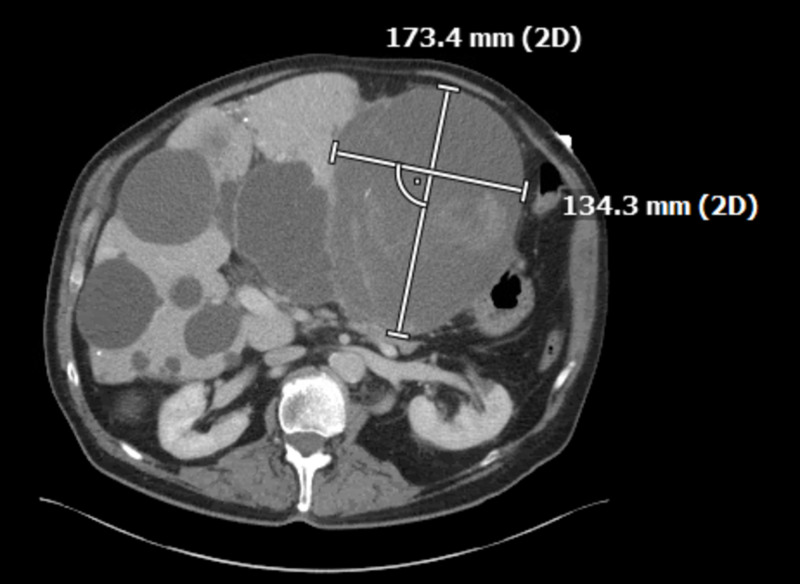
Left hepatic cyst, measuring 17.3x13.4 cm, one week prior to presentation

Multiple cystic lesions were identified throughout the liver, consistent with acquired cystic disease. Some of these lesions demonstrated peripheral calcifications within the right lobe. The lesion, a large left hepatic cystic lesion, measured 18.9x15 centimeters (Figures 3-4) and had previously measured 17.3x13.4 centimeters (Figure 5). An area of active extravasation was seen in its posterior aspect from a branch of the accessory left hepatic artery. Additionally, hemoperitoneum was noted. Bilateral pleural plaque calcifications, greater on the right than the left, suggested asbestos-related pleural disease. The interval increase in size of the left hepatic lesion with worsening hemorrhage and an area of active extravasation likely accounted for the patient’s syncopal episode.

During the ED stay, the patient became diaphoretic, tachycardic, and increasingly uncomfortable. His systolic blood pressure dropped into the low 90s. Intravenous vitamin K and factor eight inhibitor bypassing activity (FEIBA) were given to reverse the effect of rivaroxaban. His hemoglobin dropped to 8.6 g/dl from the previous value of 13.5 g/dl a week prior.

An urgent IR consultation was obtained for angioembolization of the intra-hepatic bleed within the hepatic cyst. The right common femoral artery was accessed using a 5-French micropuncture set under direct ultrasound visualization. The procedure involved the placement of a short 5-French sheath.

**Figure 6:**
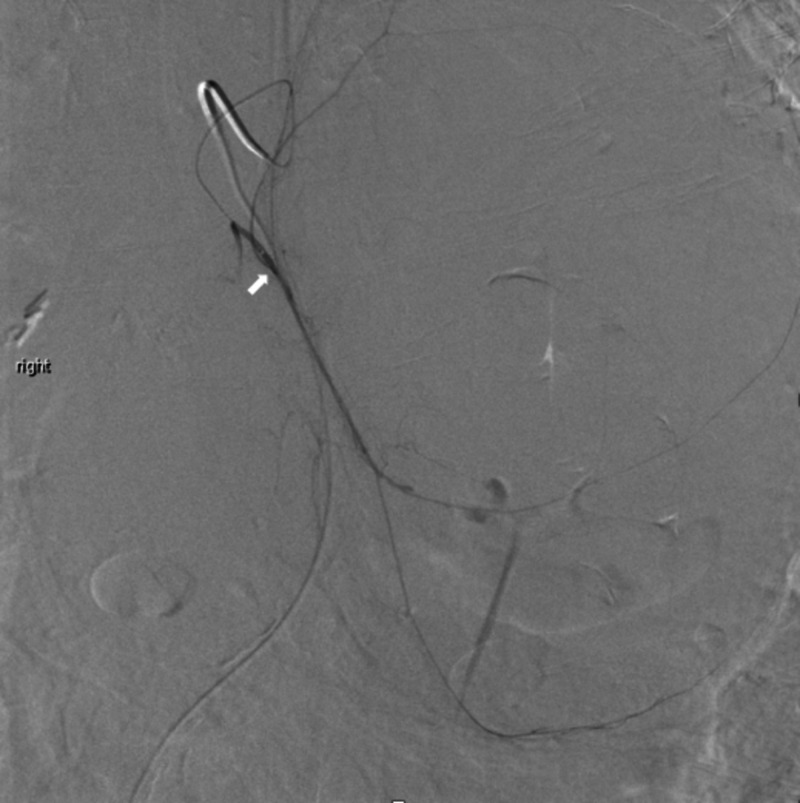
Pre-embolization of the left hepatic artery

**Figure 7:**
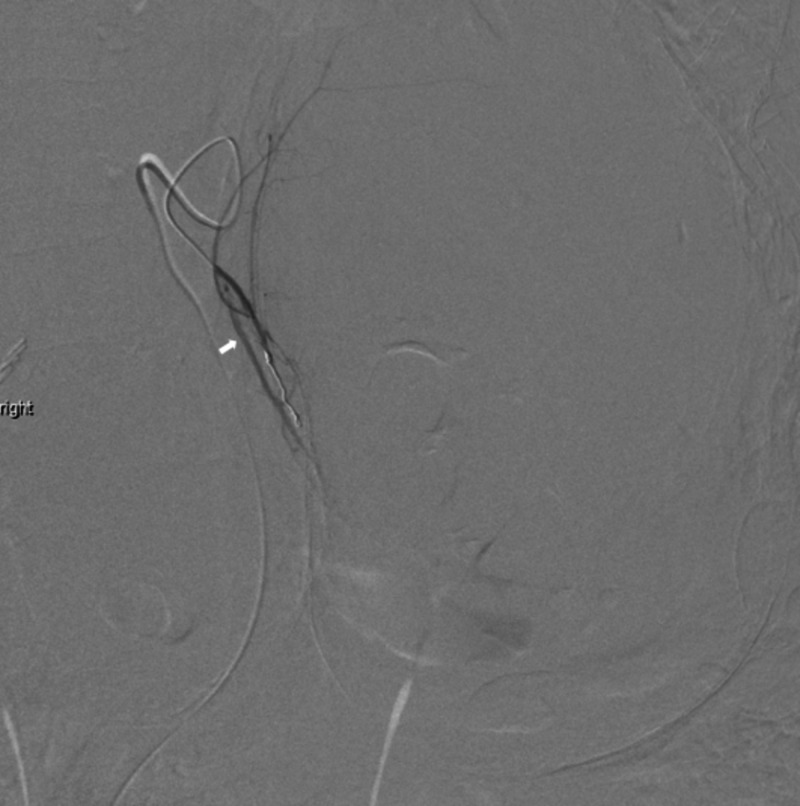
Post-embolization of the left hepatic artery

A 5-French Mickelson catheter (Cook Medical Inc., Bloomington, IN) was introduced through femoral access to catheterize the celiac artery. Contrast imaging demonstrated a very small left gastric artery. Using a 2.8-French Terumo Progreat Omega microcatheter (Terumo Medical Corporation, Somerset, NJ), the left hepatic artery was selectively catheterized. This was subsequently found to be the middle hepatic artery, though it arose approximately 1 cm distal to the takeoff of the gastroduodenal artery. Selective catheterization of this artery was performed. Though no contrast extravasation was identified, embolization utilizing polyvinyl alcohol (PVA) particle sizes of 355-500 microns (Boston Scientific, Marlborough, MA) was performed. Next, the main left gastric artery was identified as arising directly from the abdominal aorta just above the celiac artery, and selective catheterization was performed. Contrast injection and imaging delineated the left branches of the stomach and also the replaced left hepatic artery. Selective catheterization of the replaced left hepatic artery was performed, which clearly identified the site of active bleeding. Next, super-selective catheterization of the segmental branch to the area was performed. After confirmation of suitable positioning, PVA particle embolization was performed using PVA particles ranging in size from 355 to 500 microns (Boston Scientific). Lastly, a single 2 mm x 4 cm Azur-18 helical hydro coil (Terumo Medical Corporation) was deployed. A final post-embolization image confirmed the successful resolution of the bleeding site (Figures 6, 7).

The patient tolerated the procedure well and remained hemodynamically stable. The left hepatic artery is shown pre- and post-embolization in Figures 6, 7. He was subsequently transferred to the hepatobiliary/liver transplant service, where he underwent conservative supportive management for the next several days. The patient’s care was discussed in a multidisciplinary forum with hepatology, nephrology, transplant coordinators, pharmacy, and social work. The patient was discharged on hospital day four. He was followed up in the clinic at two weeks and four weeks following discharge. He remained asymptomatic and comfortable.

## Discussion

Intra-hepatic cysts are grossly classified as congenital, traumatic, infectious, parasitic, or neoplastic. Congenital hepatic cysts are further classified into simple hepatic cysts and adult polycystic liver disease [4].

Polycystic liver disease most commonly occurs with autosomal dominant polycystic kidney disease (ADPKD). Isolated PCLD is extremely rare, with incidences ranging from 1/100,000 to 1/1,000,000 [2]. Hepatic cysts are primarily asymptomatic, but advanced PCLD can present with pain, abdominal discomfort, and early satiety, depending on the size and number of cysts [11]. Though uncommon, complications such as infection, rupture, and intra-cystic bleeding can occur [10]. Compression of the inferior vena cava (IVC) leading to obstruction of venous return or thrombosis has been reported [4]. Our patient presented with diffuse abdominal tenderness and progressive distension. The patient’s syncopal episode that precipitated his second ED visit was likely due to the worsening hemorrhage.

Hepatic cysts are usually found on imaging, either with ultrasound, CT, or MRI [2]. Hemorrhagic hepatic cysts can mimic biliary cystadenoma or cystadenocarcinoma [4]. In this patient, CT was performed upon presentation to the ED because of his abdominal pain and distension, but as they are often asymptomatic, many hepatic cysts are found incidentally. 

Hemorrhagic rupture of a hepatic cyst secondary to trauma is an exceedingly rare complication of simple hepatic cysts, and very few cases have been described in the literature. Reported cases of ruptured cysts have had dimensions larger than 10 cm [11]. There is no clear consensus as to the most appropriate management strategy, but conservative management, IR embolization, and surgical intervention have all been utilized [12].

Some surgeons prefer surgery as the primary interventional modality, especially in young and healthy patients. Some of the common procedures include partial hepatectomy, cystectomy, and fenestration (laparoscopic or open), especially in those where malignancy cannot be ruled out with certainty [4]. While the former two procedures can be curative, fenestration is a relatively simpler procedure that can produce therapeutic benefits and acceptable long-term results with minimal morbidity [13, 14].

In certain cases of open emergency surgery, occlusion of hepatic inflow and intrahepatic IVC are paramount for the control of hepatic or retrohepatic IVC bleeding. Total hepatic vascular exclusion (THVE) may be applied in such a situation [1]. In patients with significant comorbidities, e.g., ischemic heart disease, heart failure, pulmonary hypertension, and cardiomyopathy, veno-venous bypass (VVB) may be helpful to avoid hemodynamic instability from IVC cross-clamping [15, 16]. Although it is imperative to maintain intraoperative hemodynamic stability, in order to prevent postoperative pulmonary edema and ascites, over-transfusion is essential to avoid [1]. Although liver transplant literature describes 1.5-5.0 L/min shunt flows [1], normovolemic stability and renal perfusion can be achieved with a shunt flow of greater than 3.0 L/min during cross-clamping of the IVC [17].

Ill-conditioned elderly patients with multiple comorbidities, especially on anticoagulation, benefit from selective or super-selective transcatheter arterial embolization. Other non-invasive procedures such as transhepatic cyst drainage and transcystic ethanol injection have also been practiced [4], though with a higher bleeding recurrence rate [18, 19].

Our patient was on novel anticoagulation medication (INR of 4.2) and therefore required urgent reversal with vitamin K and FEIBA when he became hemodynamically unstable. Due to his age and comorbidities, IR intervention was chosen as the optimal treatment option.

The patient’s CTA showed an actively hemorrhaging left hepatic cyst that was larger than on his previous ED admission. Further complicating this case, the patient had a completely replaced left hepatic artery arising from the left gastric artery, which branched directly from the abdominal aorta. On CTA, it had appeared that the patient had an accessory hepatic artery from the gastric artery, a normal anatomic variant, but upon contrast injection during the embolization procedure, it was demonstrated that it was actually a replaced left hepatic artery originating from the left gastric artery. There was evidence with selective angiography of active bleeding from the lateral left lobe of the liver, segment III. Following embolization of the replaced left hepatic artery, the resolution of the bleeding was confirmed with post-embolization imaging. As only 55% of people have the most commonly described hepatic vasculature pattern [20], one important consideration when identifying a hemorrhage is whether or not vessels can be accurately identified with CT angiography alone. In cases where surgical intervention may be a desirable option, anomalous hepatic vasculature could cause unexpected complications if not identified preoperatively on CT angiography.

## Conclusions

Traumatic hemorrhagic rupture of hepatic cysts is a rare complication of PCLD and potentially fatal if not addressed promptly. Hemorrhage complicating one of the multiple cysts has rarely been reported. In our case, the hemorrhage was successfully managed using coil embolization after identifying a replaced left hepatic artery responsible for the bleeding. There are multiple treatment modalities that can be used, but because of the small number of reported cases and treatment outcomes to evaluate, a clear consensus on the best treatment plan is lacking. We found IR embolization to be the best option for our patient.
